# Family medicine in Denmark: Are there lessons for Botswana and Africa?

**DOI:** 10.4102/phcfm.v8i1.1026

**Published:** 2016-03-30

**Authors:** Vincent Setlhare

**Affiliations:** 1 Department of Family Medicine and Public Health, Faculty of Medicine, University of Botswana, Botswana

## Abstract

Family medicine is a new specialty in Botswana and many African countries and its definition and scope are still evolving. In this region, healthcare is constrained by resource limitation and inefficiencies in resource utilisation. Experiences in countries with good health indicators can help inform discussions on the future of family medicine in Africa. Observations made during a visit to family physicians (FPs) in Denmark showed that the training of FPs, the practice of family medicine and the role of support staff in a family practice were often different and sometimes unimaginable by African standards. Danish family practices were friendly and enmeshed in an egalitarian and efficient health system, which is supported by an effective information technology network. There was a lot of task shifting and nurses and clerical staff attended to simple or uncomplicated aspects of patient care whilst FPs attended to more complicated patient problems. Higher taxation and higher health expenditure seemed to undergird the effective health system. An egalitarian relationship amongst patients and healthcare workers (HCW) may help improve patient care in Botswana. Task shifting should be formalised, and all sectors of primary healthcare should have fast and effective information technology systems. HCW training and roles should be revised. Higher health expenditure is necessary to achieve good health indicators.

## Introduction

Family medicine (FM) is at a developmental stage and it is still a new discipline in Botswana and most African countries.^1,2^ In Botswana and other African countries, FM is still defining itself, grappling with how to increase and efficiently use its human resources, integrate various vertical programmes, as well as deal with a challenging disease profile.^2,3^ In this region, FM and primary healthcare (PHC) are implanted in limited health resources; economic, political and geographical difficulties; inequalities in healthcare; gender inequality; as well as the extended family.^4,5^ Health systems vary from country to country. Uganda has a six-tier system, with lay health workers at the bottom and the national referral hospital at the pinnacle.^6^ Some even advocate for two types of African family physicians (FPs): hospital based or clinic based.^7^ The duration of training for FP varies from 2 years in Sudan^8^ to 4 years in Southern Africa. There are differences as to where FPs should be deployed, and congruence in their job description is not complete.^5,9^ Thus, African FM has country-specific characteristics that are determined by contextual challenges.^4,7^ Discussions on FM theory, practice and training should continue so as to reach a common understanding.

Research of the African context by Africans is key^10^ in ensuring that FM develops such that it becomes the backbone and main thrust of efficient healthcare.^11^ However, total exclusion of experiences from other regions would be foolhardy.

During a visit to Denmark, in March 2015, the practice and teaching of FM medicine in that country were noted and recorded. The article attempts to share observations of Danish FM practice and training and to discuss implications for Botswana and Africa.

There were differences between Botswana and Danish PHC systems; FPs, nurses, practice clerks, patients related in an egalitarian manner; task shifting facilitated Danish PHC efficiency; Danish PHC was supported by a reliable modern Information Technology (IT) system; and the training of Danish FM physicians is different and longer than that in Southern Africa.

## The Danish primary healthcare service

The top tax rate is 25% in Botswana,^12^ but Danes are taxed up to 60% to support their welfare state. PHC services are provided free of charge by the Danish government, whilst in Botswana, citizens are supposed to pay a small fee, and these policies help improve access and utilisation, positively impacting health indicators.^13^ Denmark is 20 times richer than Botswana, has only two and a half times the population, and unlike Botswana, it has no people living in poverty (Table 1). These factors together with lower budget allocations to health expenditure, and the comparatively very high income gap in Botswana (Gini Index 0.5)^14^ contribute to poorer health indicators of Botswana (Table 2).

**TABLE 1:** Economic indicators (World Bank).^15,16^

Indicator	GDP, in billion (US$)	GNI per capita (US$)	Population, in million	% poor in population
Botswana^16^	15.81	7240	2.22	19.3
Denmark^15^	342	61 310	5.64	0

*Source*: World Bank Group (2015)

**TABLE 2:** Health indicators (World Health Organization).^17,18^

Indicator	Life expectancy at birth (2013)	Maternal mortality ratio (per 100 000 live births) in 2013	% government budget expenditure on health (2013)
Botswana^18^	64	170	8.8
Denmark^17^	80	5	15.9

*Source*: World Health Organization (2015)

Lower taxes in Botswana may be contributing to frequent unavailability of drugs and equipment in public health facilities, but this could be alleviated in Botswana and African countries if 15% of their budgets were allocated to health.^19^ Botswana currently spends around 5.4% of its GDP on health, whilst Denmark spends twice that amount.^20^

Most Danes use the public health sector, although there are a few private health facilities. Danes register with a FP who attends their medical problems and they are free to change FPs at not too frequent intervals. In Botswana, patients receive western medical care mostly in the public sector but a sizable number use the private health sector.^21^

Denmark has rural-urban inequalities in the distribution of FPs such that urban areas have a disproportionately higher number of FPs. One FP attributed this to the fact that there are more women graduating as FPs than men, and these female FPs associate with professionals who cannot find suitable jobs for themselves in rural areas. A similar female predominance in undergraduate and postgraduate healthcare worker (HCW) trainees in Botswana may sustain the existing rural-urban inequalities in HCW distribution.^22^ Lack of amenities and other factors make rural practice unattractive for HCWs in Botswana and other developing countries.

## The family physicians in the Danish primary healthcare system

Only qualified FPs work in clinics, and this contributes to the good health indicators that Denmark has. In Botswana non-specialist doctors practise independently, and this may compromise care but plans are underway to make specialisation a requirement for private practice but it seems there are no such plans for the public sector. The shortage of doctors makes extended training of doctors to specialist level a challenge in resource-limited settings like Botswana.

Approximately 30% of a Danish FP’s salary is guaranteed. The rest of the salary is proportional to the number of patients the FP consults. Botswana could consider paying doctors a bonus based on the number of patients they attended to. This may increase access and utilisation of health services by increasing the numbers of patients seen. However, unlike Botswana, Denmark has a doctor-to-population ratio of 34.2 per 10 000 population,^17^ whilst according to Nkomazana et al, Botswana’s doctor-to-patient ratio is only 3.4 per 10 000 population.^23^ It would therefore be prudent to do a cost-benefit analysis of paying doctors a bonus based on the number of patients they have consulted, before implementing such an intervention.

Danish FPs act as gatekeepers to the rest of the health system. This improves efficiency of the health system, which likely contributes to the enviable health indicators of Denmark.^24^ Botswana’s referral system is still developing, and patients use the various levels of healthcare in a less orderly manner. This negatively impacts Botswana’s overburdened and under-resourced health system.

The Danish FP has ultimate responsibility for patients in his care and this promotes continuity of care, gate keeping and efficiency in healthcare delivery. Registration of patients with a FP, doctor or clinic needs to be tried in Botswana. The challenge in Botswana is that people often move between three residences (village/town, fields and cattle post/ranch). This is compounded by internal and external migration of many HCWs. Lack of a robust IT system also makes it difficult to ensure continuity of care and preservation of patient records.

Migration of doctors from the public sector to the private sector worsens doctor-to-patient ratios in the public sector, the sector that serves most patients, in Botswana.^21^ This contributes to inefficiencies in the public health sector, making patients to seek healthcare in the private sector at costs that they cannot sustain. This leads to unsatisfactory patient care. Universal health coverage that is based on public-private partnership may help optimise access and utilisation of all health resources in the country.

### Training of family physicians

FP training in Denmark includes an initial 6 months of FM residency in a FP-run clinic. This is then followed by 6 months in another FP-run clinic where formal training in FM begins. Subsequently, there are rotations of 6 months each in internal medicine, paediatrics, obstetrics/gynaecology, surgery and psychiatry. During these rotations, residents go back to a FM clinic once a month for FM tutorials. On completion of all these rotations, residents do 6 months of FM training in a clinic. The last leg of training is a 12-month stint in a clinic working almost independently. Alongside these 5 years of clinical training, there is a theoretical course that runs parallel with rotations where residents in a region are brought together for a few days, periodically. These theoretical modules include a research training module that is completed by doing a small research project, but writing a thesis is not mandatory. There is no exit examination because accreditation is based on progress assessed and ratified by clinical tutors.

The University of Botswana 4-year postgraduate FM programme meets the needs of Botswana. The requirements of research training and research practice with journal article or dissertation writing may be worth considering by the Danes.

### The laboratory services

FPs, nurses and practice clerks collect specimens, which are collected twice a day. Laboratories avail results online within 12–24 hours, ensuring safety of records and improving patient care. Collecting specimens in PHC facilities, getting specimens to laboratories, getting specimens processed and accessing laboratory results is often a challenge in Botswana and this is compounded by lack of a functional IT network. North-South collaboration in building a robust IT system in the Botswana health system would benefit patient care.

### Specialists as ordinary people

FPs were relaxed and informally dressed like their patients, clerks and nurses. FPs treated each other, patients, clerks and nurses as equals and many FPs had tea and lunch with their staff which may be conducive to building strong teams.

Botswana inherited a hierarchical medical system but the nursing leadership is more American oriented and more informal. This has often led to friction between nurses and doctors in defining who is in charge and in sharing responsibilities in patient care. Resolution of these challenges and a more egalitarian paradigm would enhance patient care.

### The consultation

The length of a consultation differed according to varying patient needs, but FPs consulted for 5–10 minutes on average (and this included taking specimens and doing minor procedures). Patients seemed happy with this, and safe netting ensured that serious illnesses were not missed. The length of the consultation was encouraging to observe because junior residents in Botswana seem more comfortable with 30- to 40-minute consultations, which are usually out of place in busy, understaffed primary care clinics.

### Well organised, homely clinics

The clinics visited in Denmark were in a residential area and looked like other residences in the area. The clinics were clean and had a homely ambience. The reception area had functioning telephones and computers. The clerks handled calls with courtesy, knowing what to do and taking decisions quickly. The interaction between FPs, clerks, nurses, patients and couriers of laboratory specimens seemed easy and seamless. Quite often in Botswana, authority/hierarchy-related friction between HCWs makes healthcare delivery difficult and this may be because of ill-defined roles.

### Roles of clerks and nurses

Clerks were the first point of contact with patients, and the clerks had no formal medical education, were pharmacists or had a nursing background. They were previously trained by individual clinics, but since 2009, clerks undergo a formal 2-year course for practice clerks.

Clerks triaged patients, deciding if a patient was going to be treated by a clerk, nurse (for minor ailments) or FP. Patients treated by a clerk or nurse could be availed an appointment with a FP if they did not improve. Clerks also did electrocardiograms, phlebotomies for specimen collection, took urine specimens, did acupuncture, gave lifestyle advice and took blood pressures.

In Botswana, janitors often take patients’ weights, blood pressures and temperatures and record these, whilst pharmacists and nurses sometimes do pap smears, consult with patients and take specimens for laboratory investigations. In Botswana, these arrangements are informal and are often a cause of friction amongst HCWs when colleagues choose not to cooperate.

The ethical implications of risking patients’ lives by shifting medical tasks to untrained workers (janitors) are disturbing and are yet to be addressed in the Botswana clinics. Trained HCWs making health-related decisions based on information from untrained workers should be a cause for concern.

In Denmark, chronic patients who are healthy are not routinely seen by a FP unless the patient has a problem or is scheduled for a check-up with the FP. PHC in Botswana would be more efficient if healthy, chronic patients consulted with nurses and pharmacists. Many healthy people with HIV and/or AIDS, patients with diabetes, patients with tuberculosis and hypertensive patients are seen by doctors every visit, leaving doctors hard pressed for time to attend to very ill patients.

### Nursing homes

In Denmark, elderly peolpe who are ill, cannot take care of themselves and have no relative to look after them are offered accommodation in nursing homes where they are taken care of by nurses, providing them care and medications. These elderly people are availed services of their FPs in the nursing home, when necessary.

Botswana still has the extended family taking care of the elderly, though this institution is undergoing strain. Home-based care, within the Botswana public health system, should be strengthened so that it may empower the extended family to take care of the elderly.

## Conclusion

Unlike Danish society, African societies are largely hierarchical,^25^ and some have inherited hierarchical medical systems. Hierarchical health systems probably compromise patient care by stifling views that challenge experts or seniors. The Danish model provides an egalitarian alternative that enables HCWs in their different disciplines and levels of expertise to provide a largely efficient healthcare delivery system. Together with appropriate government health financing, good doctor-to-patient ratios, task shifting and connecting the various arms of the health sector with reliable and fast IT, the amicable work environment facilitates achievement of admirable health indicators for the Danish nation.^24^

Task shifting or competency shifting is essential in under-resourced countries so as to increase efficiency, effectiveness, access and utilisation of health services.^26^ However, competency shifting becomes a source of friction where it is not formalised or where it is not accepted by all HCWs. Task shifting should be formalised in the Botswana health service so as to improve access and utilisation of healthcare.^26^

Clinics and hospitals are strange and intimidating to patients. The Botswana government should stop constructing new clinics and consider buying residences and converting them into clinics where patients could feel relaxed and ‘at home’. This may enhance patient care by enabling patients to consult freely and fully in familiar environments.

The efficient Danish health system is undergirded by a reliable IT system and Botswana should invest in a single, fast, efficient IT system across the different sectors of the health system. HCWs should be computer literate, and health facilities should have IT technicians on site to ensure that the IT system is working.

In Botswana, we should rethink the need and training of different HCWs and discontinue producing HCWs based on tradition. HCWs should be trained for tasks and sectors in which they will serve. Hospitals and clinics serve different purposes and require HCWs with different tasks and skills. Danish and Botswana clinics also show that many health-related tasks can be carried out by workers with no nursing, medical or pharmaceutical background.

In this age of rapid tests and meagre resources, medical scientist and technician training should be reviewed. FPs, doctors, nurses and support staff can do many rapid tests and obtain results immediately and this increases efficiency in PHC. An overloaded health system like that in Botswana should tap into these technologies and tailor the training of laboratory HCWs to fit the new paradigm.

Botswana and other African nations should adhere to the Abuja Declaration, which states that 15% of their budgets should be allocated to health. Failure to do this makes it more difficult to improve the health of communities in these countries.

The egalitarianism in the Danish health system, substantial health expenditure, an effective and reliable IT system, task shifting and the staffing of clinics with specialist FPs have contributed to the achievement of admiral health indicators for Denmark. There may be useful lessons for Botswana and Africa to learn from the Danish model.

## References

[1] Mash B, Downing R, Moosa S, De Maeseneer J Exploring the key principles of Family Medicine in sub-Saharan Africa: international Delphi consensus process. S Afr Fam Pract J. 2008;50(3):60–65.

[2] Monjok E, Okokon IB, Smesny A, Essien EJ Rural Health and Family Medicine: an Agenda for sub-Saharan Africa. Afr J Prim Health Care Fam Med. 2011;3(1):2.

[3] De Maeseneer J Scaling up Family Medicine and Primary Health Care in Africa: statement of the primafamed network Victoria Falls, Zimbabwe. Afr J Prim Care Fam Med. 2013;5(1):3.

[4] Mash R, Reid S Statement of consensus on Family Medicine in Africa. Afr J Prim Care Fam Pract. 2010;2(1):4.

[5] Ministry of Health Kenya. Proposed Family Medicine Policy. Nairobi, Kenya: Government of Kenya, 2007.

[6] Ssenyonga R, Seremba E Family Medicine’s role in Health Care Systems in Sub-Saharan Africa: uganda as an example. Fam Med. 2007;39(9):4. 17932794

[7] Besigye IK, Namatovu JF, Scaling up Family Medicine in Uganda. Afr J Prim Health Care Fam Med. 2014;6(1):3. 10.4102/phcfm.v6i1.664 PMC5337989 26245446

[8] Mohamed KG, Hunskaar S, Abdelrahman SH, Malik EM Scaling up family medicine training in Gezira, Sudan – a 2-year in-service master programme using modern information and communication technology: a survey study. Hum Resour Health. 2014;12(3):9. 24443978 10.1186/1478-4491-12-3 PMC3900464

[9] Ministry of Health Botswana. Job description – consultant family physician. Gaborone, Botswana: Ministry of Health Botswana p. 2.

[10] Setlhare V, Couper I, Wright A Patient-centredness: meaning and propriety in the Botswana, African and non-Western contexts. Afr J Prim Care Fam Med. 2014;6(1):4. 10.4102/phcfm.v6i1.554 PMC5337990 26468553

[11] WHO. Declaration of Alma-Ata International Conference on Primary Health Care. Alma-Ata, USSR: WHO: 1978.

[12] Botswana Unified Revenue Service. Tax tables and guidance notes. Gaborone, Botswana: Government of Botswana, 2011 p. 84.

[13] WHO. Flawed but fair: brazil’s health system reaches out. Bull World Health Org. 2008;86(4):79. 10.2471/BLT.08.030408 PMC2647429 18438510

[14] International Monetary Fund. IMF country report no. 12/235: Botswana. Washington, DC: International Monetary Fund Publication Services, 2012 p. 44.

[15] The World Bank. Data> countires and economies> Denmark. Washington, DC: The World Bank Group, 2015.

[16] The World Bank. Data> countries and economies> Botswana. Washingoton, DC: The World Bank Group, 2015.

[17] World Health Organization. Denmark statistics summary (2002 – present). Global Health Observatoy country views. Geneva, Switzerland: World Health Organization, 2015.

[18] World Health Organization. Botswana statistics summary (2002 – present). Global Health Observatory country views 2015. Geneva, Switzerland: World Health Organization, 2015.

[19] Organization of African Unity. Abuja declaration on HIV/AIDS, tuberculosis and other related infectious diseases. Abuja, Nigeria: Organisation of African Unity, 2001 p. 1–8.

[20] The World Bank. Health expenditure, total (% of GDP). World Bank Group, 2015.

[21] Government of Botswana. Republic of Botswana Health Statistics Report 2008. Gaborone: Statstics Botswana, 2012.

[22] Munga MA, Maestad O Measuring inequalities in the distribution of health workers: the case of Tanzania. Hum Res Health. 2009;7(4). 10.1186/1478-4491-7-4 PMC2655278 19159443

[23] Nkomazana O, Peersman W, Willcox M, Mash R, Phaladze N Human resources for health in Botswana: the result of in-country database and reports analysis. Afr J Prim Health Care Fam Med. 2014;6(1). 10.4102/phcfm.v6i1.716 PMC4564932 26245420

[24] QUANDL. Denmark health data. QUANDL, 2012.

[25] Amanze J Witchcraft beliefs and practices, In: Amanze J., editor African traditional religions and culture in Botswana. Gaborone, Botswana: Pula Press, 2002 p. 233–245.

[26] World Health Organization. Task shifting: global recommendations and guidelines Guidelines. World Health Organization, editor Geneva, Switzerland: WHO Press, 2008.

